# Small interfering RNAs in the management of human osteoporosis

**DOI:** 10.1093/bmb/ldad023

**Published:** 2023-09-06

**Authors:** Giuseppe Gargano, Giovanni Asparago, Filippo Spiezia, Francesco Oliva, Nicola Maffulli

**Affiliations:** Department of Trauma and Orthopaedic Surgery, AOU San Giovanni di Dio e Ruggi D’Aragona, Via San Leonardo 1, 84131 Salerno, Italy; Department of Medicine, Surgery and Dentistry, University of Salerno, Via S. Allende, 84081 Baronissi (SA), Italy; Department of Trauma and Orthopaedic Surgery, AOR San Carlo, Via Potito Petrone, 85100 Potenza, Italy; Department of Trauma and Orthopaedic Surgery, AOU San Giovanni di Dio e Ruggi D’Aragona, Via San Leonardo 1, 84131 Salerno, Italy; Department of Medicine, Surgery and Dentistry, University of Salerno, Via S. Allende, 84081 Baronissi (SA), Italy; Department of Trauma and Orthopaedic Surgery, AOR San Carlo, Via Potito Petrone, 85100 Potenza, Italy; Department of Trauma and Orthopaedic Surgery, AOU San Giovanni di Dio e Ruggi D’Aragona, Via San Leonardo 1, 84131 Salerno, Italy; Department of Medicine, Surgery and Dentistry, University of Salerno, Via S. Allende, 84081 Baronissi (SA), Italy; Queen Mary University of London, Barts and the London School of Medicine and Dentistry, Centre for Sports and Exercise Medicine, Mile End Hospital, 275 Bancroft Road, London E1 4DG, UK; School of Pharmacy and Bioengineering, Keele University School of Medicine, Thornburrow Drive, Stoke on Trent, UK; Department of Orthopaedic Surgery and Traumatology, University of Rome La Sapienza, Hospital Sant’Andrea, Rome, Italy

**Keywords:** osteoporosis, osteoporosis therapy, small interfering RNA, short interfering RNA, RNA silencing, RNA interference

## Abstract

**Background:**

Osteoporosis results in reduced bone mass and consequent bone fragility. Small interfering RNAs (siRNAs) can be used for therapeutic purposes, as molecular targets or as useful markers to test new therapies.

**Sources of data:**

A systematic search of different databases to May 2023 was performed to define the role of siRNAs in osteoporosis therapy. Fourteen suitable studies were identified.

**Areas of agreement:**

SiRNAs may be useful in studying metabolic processes in osteoporosis and identify possible therapeutic targets for novel drug therapies.

**Areas of controversy:**

The metabolic processes of osteoporosis are regulated by many genes and cytokines that can be targeted by siRNAs. However, it is not easy to predict whether the *in vitro* responses of the studied siRNAs and drugs are applicable *in vivo*.

**Growing points:**

Metabolic processes can be affected by the effect of gene dysregulation mediated by siRNAs on various growth factors.

**Areas timely for developing research:**

Despite the predictability of pharmacological response of siRNA *in vitro*, similar responses cannot be expected *in vivo*.

## Introduction

Osteoporosis (OP) is a common metabolic bone disease, with a higher incidence in the elderly and postmenopausal population.^1^ Affected patients develop a reduction in bone mass with consequent bone fragility. The bone microarchitecture is altered from an imbalance of function between osteoclasts and osteoblasts.^1^ In particular, the increased osteoclastic activity causes fragility that predisposes to fractures after even minimal trauma.^1–3^ Advancing age is a predisposing factor, but it is not the cause of osteoporosis.^4–6^ Physiologically, in elderly subjects the activity of osteoclasts tends to be greater than that of osteoblasts. In osteoporosis, the activity of the osteoclasts produces excessive resorption, which therefore exceeds the physiological aging of the bone.^5^^,^^7^

The current management of OP aims to re-integrate bone components through the use of calcium, vitamin D; hormones or drugs that act on osteoclastic activity can be used, but the results are often unpredictable, and undesirable side effects are often encountered.^5^^,^^8^

Recent scientific research has focused on the regulatory mechanisms of eukaryotic cells,^9–11^ including ribonucleic acid interference (RNAi),^12–15^ to identify possible molecular and gene targets to formulate novel therapies.^16–19^

Usually, a small interfering RNA (siRNA) is composed of about 20 nucleotides arranged to form a double-stranded ribonucleic acid (RNA) molecule.^12^^,^^20^^,^^21^

**Fig. 1:**
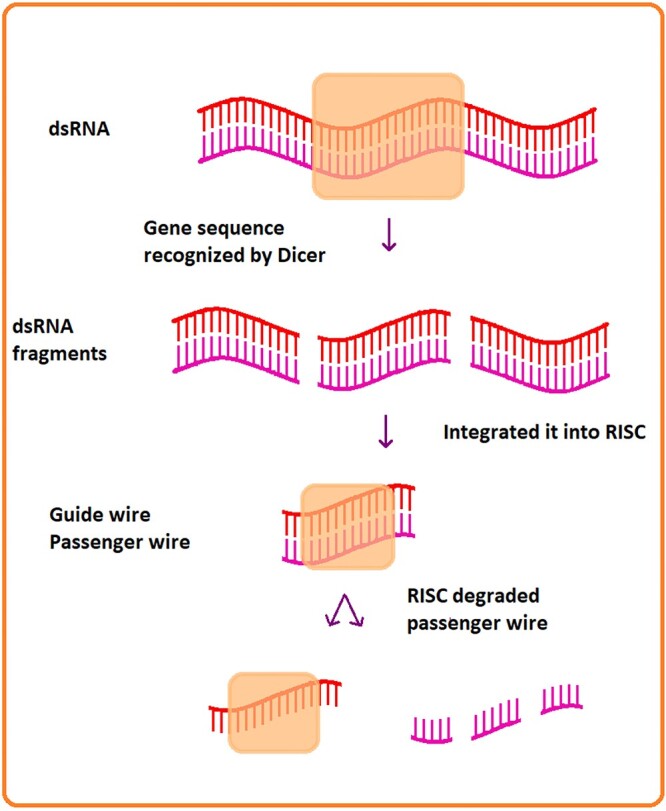
Mechanism to degrade the messenger wire.

The interference mechanism through which RNAi acts involves various elements, such as detection wire (passenger wire), sense wire (guide wire), enzymes such as Dicer, Argonaute and the central part RISC (RNA-induced silencing complex). The guide wire is a nucleotide sequence recognized by Dicer, which selects it and integrates it into RISC. The guide wire is used to recognize the passenger wire, which will be then degraded by RISC^12^^,^^22^ (Figure 1).

The study of siRNA should allow to understand their physiological role, and consequently use their activity to modulate it for therapeutic purposes. The field of application of siRNA is very varied, and gene therapies can be used for viral infections, autoimmune diseases or tumors and endocrinological diseases.^12^^,^^22^ The use of siRNA can reduce the expression of genes involved in several conditions. To date, the sequence of 4894 chemically modified siRNAs is available.^13^^,^^23^ SiRNAs can be used to study human pathologies and the biological processes involved in such pathologies. However, they have a short half-life. Structural chemical modifications are used to increase the half-life of siRNAs, making them more stable.^12^^,^^22^

In OP, the imbalance between bone resorption and bone apposition is determined by a decrease in the activity of osteoblasts and an increase in the activity of osteoclasts, mediated by both hormonal and molecular factors.^24^ Specific siRNAs have been used to identify specific targets for potential targeted therapies, or study specific pathways to determine factors and molecules which are increased and decreased in OP.^24^

The present review evaluates the current scientific evidence on the use of siRNAs in the management of osteoporosis.

## Methods

**Fig. 2:**
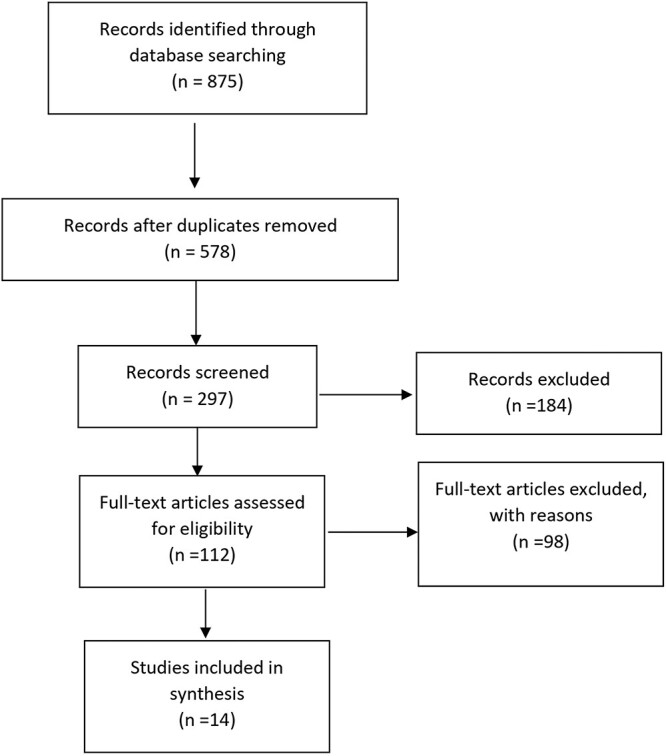
PRISMA flow diagram.

The review follows the Preferred Reporting guidelines for systematic reviews and meta-analyses (PRISMA)^25^^,^^26^ (Figure 2).

All published investigations reporting the possible role of siRNA in the management of OP according to *a priori* established inclusion criteria were considered.

Only studies published in English were included in the present investigation. Narrative and systematic reviews, meta-analyses, technical notes and case reports were excluded.

Two investigators independently conducted the systematic search, through May 2023, from the full-text archives of Embase, Google Scholar, Scopus and PubMed. In the search, we used combinations of the following key terms: Osteoporosis, Osteoporosis therapy, small interfering RNA, short interfering RNA, RNA silencing, RNA interference, with no limit of year of publication. Two investigators independently examined the titles and abstracts to remove duplicates, and evaluated the eligible studies according to the pre-established inclusion criteria. If titles and abstracts did not allow to decide on inclusion or exclusion, the relevant full text was examined. The bibliographies of the articles included were reviewed by hand to identify further related articles. If discrepancies persisted, discussion with the senior investigator allowed to resolve them.

Fourteen studies satisfied the inclusion criteria, and were thus included in the analysis. The details of the search are detailed in the flowchart in Figure 1.

## Results

A total of 875 articles were identified. The duplicates were subsequently removed, obtaining 578 articles. At this point, 297articles were excluded after reading the titles and abstracts. Of the remaining 112 articles, 98 were excluded as they were not appropriate for the topics covered or for the incomplete amount of information reported.

Data from the 14 studies which met the inclusion criteria were extracted and collected in Table 1.

**Table 1:** Studies included

**Study**	**siRNA Target Gene** ^*^	**Function on osteoporosis therapy**	**Drugs activity tested**	**Cells analyzed**	**Type of study**
Tang et al. 2012^32^	PTDINS (3, 4, 5) P3	PtdIns(3,4,5)P3 inhibits the osteogenetic response through specific mediators	ARAP3 siRNA	Mesenchymal stem cells (MSC)	in vitro
Zhu et al. 2012^33^	EGFR and amphiregulin	Amphiregulin-EGFR Signaling Mediates the Migration of Bone Marrow Mesenchymal Progenitors toward parathyroid hormone (PTH) stimulated osteoblasts and osteocytes	EGFR-siRNA and amphiregulin siRNA	Bone Marrow Mesenchymal Progenitors	in vitro
Yang et al. 2013^30^	knockdown of FOXO3A with siRNA	Forkhead box O (FOXO) transcription factors acts as an important defense mechanism against oxidative stress	Tanshinol	Mesenchymal precursor and preosteoblastic cells	in vitro
Mullin et al. 2014^34^	RHOA and ARHGEF3	The concentration of RHOA and ARHGEF3 genes change in bone mineral density in postmenopausal women	RHOA siRNA and ARHGEF3-siRNA	Osteoblast and osteoclast	in vitro
Sun et al. 2015^35^	CNR2	CNR2 promoted expression of osteogenic genes and enhanced deposition of calcium in extracellular matrix	CNR2-siRNA	Bone marrow mesenchymal stem cells (BM-MSCs)	In vitro
Tong et al. 2015^36^	DANCR	DANCR regulates osteoblast differentiation	DANCR-siRNA	Blood mononuclear cells (MNCs)	In vitro
Hong et al. 2016^37^	PTCH1	PTCH1 is related to Secreted Protein Acidic And Cysteine Rich (SPARC) which promotes osteogenic differentiation of stromal cells	PTCH1-siRNA	Stromal cells	in vitro
Tao et al. 2016^31^	β-catenin specific siRNA	Wingless/Integrated (WNT)/β-catenin signaling pathway is involved in osteogenic differentiation of human bone marrow-derived MSCs	Berberine (BBR)	BM-MSCs	in vitro
Liu et al. 2017^27^	PLEKHO1	Elevated PLEKHO1 levels in osteoblasts are associated with reduced bone formation during aging	PLEKHO1-siRNA	Osteoblast	in vitro
Adam et al. 2018^28^	KLF2, KLF4, CDC42, RHOA	Expression of KLF2 or KLF4 mRNA and phosphorylation of ERK5 protein induces osteogenic differentiation in MSCs	siRNAs for KLF2 and KLF4	MSC	in vitro
Bai et al. 2018^29^	GBP1	GBP1 in involved in Osteogenic Differentiation of Human Mesenchymal Stromal Cells	hGBP1-siRNA	MSCs	In vitro
Wang et al. 2018^41^	IRS2	Insulin receptor substance 2 (IRS2), binds insulin-like growth factor 1 (IGF-1) receptor tyrosine kinase, regulating osteogenic and adipogenic differentiation of MSCs	IRS2-siRNA	MSCs	In vitro
Pucci et al. 2019^42^	CLU	CLU related to osteoporosis causes a reduction in muscle mass	CLU silencing by siRNA	Myoblast	*In vivo*
Zhang 2020^43^	TGFBR1	TGFBR1 and TGFBR2 are involved in the activation and differentiation of osteoclasts	siRNA-TGFBR1 or siRNA-TGFBR2	Acute Monocytic Leukemia; Human (THP)-1 cells	In vitro

^*^Phosphatidylinositol (3, 4, 5)-trisphosphate (PTDINS (3, 4, 5) P3); ARF-GAP with RHO-GAP domain 3(ARAP 3); Epidermal Growth Factor Receptor(EGFR); Forkhead box O3a(FOXO3a); Ras homolog family member A(RHOA), Rho Guanine Nucleotide Exchange Factor 3(ARHGEF3); Cannabinoid receptor 2(CNR2); Differentiation Antagonizing Non-Protein Coding RNA(DANCR); Protein patched homolog 1(PTCH1); β-catenin; Pleckstrin homology domain-containing family O member 1 (PLEKHO1); Krüppel-like Factor 2 (KLF2), Krüppel-like Factor (KLF4), Cell Division Cycle 42 (CDC42); Guanylate Binding Protein 1 (GBP1); insulin receptor substance 2 (IRS2); Clusterin (CLU); transforming growth factor beta receptor 1 (TGFBR1).

Of these 14 studies, 12 used siRNAs to silence specific genes, and then identified gene and protein targets to produce a targeted therapy. Another two studies used siRNAs to monitor the function of some drugs used for the management of osteoporosis.

### SiRNAs as potential therapeutic agents

Liu et al.^27^ studied human osteoblasts of fractured elderly patients, and rodent osteoblasts. The concentration of pleckstrin homology domain-containing family O member 1 (PLEKHO1) increases with aging, and this is this correlated with a reduction of bone morphogenetic protein (BMP) dependent on small mother against decapentaplegic (SMAD) and bone formation. By using siRNA PLEKHO1, reducing the values of PLEKHO1 could reverse the process of bone aging. siRNA PLEKHO1 may be proposed as a possible treatment for osteoporosis.^27^

Adam et al.,^28^ using human mesenchymal stem cells (hMSC) and specific siRNA, provided evidence that nitrogen-containing bisphosphonates (N-BP) activates the mitogen-activated protein kinases cascade (MERK) 5/extracellular signal-related kinase (ERK) 5, which has an essential role in osteogenic differentiation and mineralization of skeletal precursors.^28^

Using specific siRNAs against Guanylate Binding Protein 1, Bai et al.^29^ demonstrated that the osteogenetic activity in human mesenchymal stem cells (hMSC) increased when GBP1 was inhibited, and decreased under normal conditions. This result was in line with the higher concentration of GBPs in premenopausal patients, and suggests a possible use of siRNA-GBP1 as a possible therapeutic target against osteoporosis.^29^

### SiRNAs to test the efficacy of drugs

Oxidative stress palys an important role in the progression of osteoporosis. For this reason, Yang et al.^30^ studied the effects of the natural antioxidant Tanshinol against oxidative stress on the differentiation of osteoblastic cells. Hydrogen peroxide (H_2_O_2_) leads to the accumulation of reactive oxygen species (ROS), decreased cell viability, cell cycle arrest and apoptosis in a caspase-3-dependent manner.

The action of Thansinol was tested using specific siRNAs against the transcription factor Forkhead box O3a (FOXO3A). Tanshinol suppresses the activation of FoxO3a and the expressions of its target genes.

Thansinol neutralizes the action of Growth arrest and DNA-damage-inducible protein 45 alpha (GADD45-α) and catalase (CAT), produced by DNA damage. It also counteracts the binding of Wingless (WNT) to its site of action by targeting genes for axis inhibition protein 2 (AXIN2), alkaline phosphatase (ALP), and osteoprotegerin (OPG).

Tanshinol attenuates oxidative stress through the down-regulation of FoxO3a signaling, and at least partially reverses the decrease in osteoblastic differentiation, making it a possible drug in the therapy of osteoporosis.^30^

Berberine (BBR) has recently been used in osteoporosis patients. Tao et al.^31^ investigated the osteogenic differentiation induced by this drug on bone marrow mesenchymal stem cells (BM-MSCs). For this purpose, they used β-catenin specific siRNA to study cell lines in the presence and absence of BBR.

BBR can stimulate the osteogenic differentiation of mesenchymal stem cells (MSC) by improving the expression of Runt-related transcription factor 2 (RUNX2) and activating the WNT/β-catenin signaling pathway, which is partly responsible for the osteogenic differentiation induced by MSC BBR in vitro. BBR is therefore a potential pharmaceutical drug for osteoporosis.^31^

### SiRNAs to identify potential therapeutic targets

Tang et al.^32^ studied human mesenchymal stem cells, using a specific siRNA against Alternate Reading Frame Guanosine TriPhosphatease-activating-protein (ARF-GAP) with Ras homolog Guanosine TriPhosphatease-activating-protein (RHO-GAP) domain 3(ARAP 3). They demonstrated a new pathway of osteogenic activation. siRNA ARAP3 led to the recovery of Ras homolog family member A (RHOA) and focal adhesion kinase (FAK) activities, producing an increase in osteogenic activity. This new route could be used to develop novel therapies in osteoporosis.^32^

Zhu et al.^33^ studied Bone Marrow Mesenchymal Progenitors. Stimulation with conditioned media from parathyroid hormone (PTH)-treated osteoblastic and osteocytic cells, which contain soluble chemotactic factors for bone marrow mesenchymal progenitors, resulted in increased Epidermal Growth Factor Receptor (EGFR) phosphorylation in the treated cells. The study used inhibitors, including specific siRNAs, showing that PTH increases the release of amphiregulin from osteoblastic cells, which acts on EGFRs expressed on mesenchymal progenitors to stimulate the protein-kinase B (PKB) and protein 38 mitogen-activated protein kinase (MAPK) pathways, and subsequently promote their migration *in vitro*. Subsequently, the inactivation of the EGFR signal on osteoprogenitors/osteoblasts attenuated the anabolic actions of PTH on bone formation. These results suggest a therapeutic role of PTH in osteoporosis through an anabolic effect of EGFR signaling on bone.^33^

Mullin et al.^34^ performed a knockdown study of Ras homolog (RHO) Guanine Nucleotide Exchange Factor 3 (ARHGEF3) and Ras homolog family member A (RHOA) genes using small siRNAs in human osteoblasts and osteoclast-like cells in culture. Real-Time Quantitative Reverse Transcription C-reactive Protein (QRT-PCR) showed significant down-regulation of the Actin Alpha 2 (ACT-**α**2) gene, encoding the cytoskeletal protein alpha 2 actin, in response to RHOA knockdown in both osteoblasts and osteoclasts. RHOA knockdown also upregulated the parathyroid hormone receptor 1 (PTH1R) gene. Knockdown of Rho Guanine Nucleotide Exchange Factor 3 (ARHGEF3) in osteoblast-like cells resulted in down-regulation of the Tumor Necrosis Factor Receptor Superfamily Member 11b (TNFRSF11B) gene, coding for osteoprotegerin. This study identifies ARHGEF3 and RHHOA as potential regulators genes that act in bone metabolism and can be used as targets in specific therapies for osteoporosis.^34^

Sun et al.^35^ studied the cannabinoid receptor (CNR2) on bone marrow-derived mesenchymal stem cells (BM-MSC). The study was conducted using knockdown of CNR2 by siRNA. Inactivation of the CNR2 receptor reduces the activity of alkaline phosphatase (ALP), inhibits the expression of osteogenic genes and induces a deposition of calcium in the extracellular matrix. Furthermore, bone marrow samples showed that the expression of CNR2 is much lower in patients with osteoporosis than healthy donors: CNR2 deficiency may be related to osteoporosis. In the bone marrow samples examined, the expression of CNR2 is much lower in patients with osteoporosis than healthy donors, thus raising the possibility that osteoporosis can be related to a lack of CNR2.^35^

Tong et al.^36^ used blood mononuclear cells (MNCs), as they are directly involved in osteoclastogenesis and osteoporosis. Through a specific siRNA against Differentiation Antagonizing Non-Protein Coding RNA(DANCR), they showed a reduction of interleukin 6 (IL6) and tumor necrosis factor alpha (TNF-**α**).

DANCR was therefore a regulator of the osteoblastic activity. Its inhibition induced greater osteoblastic activity, shifting the balance against osteoclastic activity thus favoring bone production and mineralization. As DANCR is overexpressed in osteoporosis, DANCR can be a target against osteoporosis.^36^

Starting from the evidence of bone abnormalities and osteoporosis in patients with nevoid basal cell carcinoma syndrome (NBCCS), Hong et al.^37^ wanted to identify a gene that could cause these effects to use targeted gene therapy in specific patients to safeguard them from the risk of osteoporosis. The identified gene, Protein patched homolog 1(PTCH1), was studied by specific siRNA. The downregulation of PTCH1 is associated with a reduction in Secreted Protein Acidic and Cysteine Rich (SPARC) expression, with a reduction in ossification. PTCH1 may be a possible target in the therapy against osteoporosis in specific patients.^37^

WNT/**β**-catenin signaling pathway decreases bone formation by reducing osteoblast differentiation.^38^^,^^39^

Many investigations have studied the differentiation of hMSCs, with an inverse relationship between adipocytic and osteocytic development. Therefore, different signaling pathways induce MSC towards osteogenic or adipocytic differentiation.^40^

Wang et al.^41^ investigated adipogenic differentiation of hMSCs by specific siRNA for insulin receptor substance 2 (IRS2). The expression of IRS2 was increased during adipogenic differentiation, but, by inhibiting it with specific siRNA, such adipogenic differentiation was inhibited.

The balance between osteogenic and adipogenic differentiation of hMSCs is altered in pathologies such as osteoporosis. Such studies may have a therapeutic value to produce drugs which block IRS2, increasing pro-osteogenic differentiation.^41^

Pucci et al.^42^ demonstrated that patients with OP exhibited degeneration of muscle fibers with an overexpression of Clusterin (CLU), correlating to high levels of IL6 and acetylation histone H4 of myoblasts. In the muscle tissues of osteoporotic patients, the muscle fibers were intact and CLU levels were low. Using specific siRNAs against CLU, inhibition of CLU restored of the ability of proliferative myoblasts and repaired muscle tissue damage. CLU could therefore be considered a potential therapeutic target in OP patients.^42^

Zhang et al.^43^ used specific siRNAs to validate data obtained through the Multiscale Embedded Gene Co-Expression Network Analysis (MEGENA) method that allows to obtain sequence of genes that are involved in the pathogenesis of osteoporosis. This allowed to identify some genes, such as transforming growth factor beta receptor 1 (TGFBR1) and transforming growth factor beta receptor 2 (TGFBR2), involved in the differentiation and recruitment of osteoclasts. This study opens up new perspectives to use siRNA to control more elaborate and large-scale pathogenetic pathways.^43^

## Discussion

Osteoporosis produces serious structural damage to bones, increases the risk of fractures, and produces deformities that can lead to bed rest and increased mortality.^1^^,^^44^^,^^45^ Osteoporosis fractures arise from multifactorial alteration of the micro-architecture of bone.^5^^,^^24^^,^^46^^,^^47^ Hormonal factors are involved. Indeed, both sexes lose bone mass during life, but after menopause women lose bone much faster and are more prone to fragility fractures. Other factors are cellular, connected to imbalances between osteoclasts and osteoblasts. Finally, calcium and vitamin D play an important role.^7^^,^^26^^,^^48–50^

Although fractures are often the first and most striking event of this pathology, such patients have developed osteoporosis long before the fracture event.^1^^,^^51^^,^^52^

Authors have performed studies on human cells, mesenchymal stem cells, Bone Marrow Mesenchymal Progenitors, osteoblasts, osteoclasts and myoblasts to investigate the various metabolic pathways and identify the molecular targets on which it may be possible to intervene.^53^

**Fig. 3:**
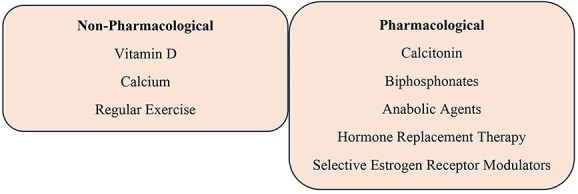
Osteoporosis treatment.

The Current management for OP is based on antiresorptive drugs, including calcitonin, oestrogens, bisphosphonates, and bone anabolic drugs, including teriparatide^1^^,^^5^^,^^8^ (Figure 3).

OP patients exhibit poor drug taking compliance. The drugs often have serious side effects and unpredictable efficacy. Among the side effects, gastrointestinal disorders are common, and the most serious, such as osteonecrosis of the mandible, occur with bisphosphonate therapy. Long term oral bisphosphonate therapy increases the risk of atypical fractures and the incidence of esophageal cancer. Therefore, treatment with bisphosphonates for no longer than five years is recommended. In 2010, denosumab, a monoclonal antibody targeting the receptor activator of nuclear factor kappa ligand (RANKL), was introduced. New therapeutic targets through the use of siRNAs can be conceived.^1^^,^^5^

Epidermal growth factor receptor (EGFR) binds to epidermal growth factor (EGF) and also to transforming growth factor **α** (TGF**α**), leading to activation of the receptor which homodimerizes with a family of proteins including human epidermal growth factor receptor 2 (ERBB- 2), human epidermal growth factor receptor 3 (ERBB-3) and human epidermal growth factor receptor 4 (ERBB-4).^54^ This type of activation induces activity of tyrosine kinase domains, resulting in phosphorylation and recruitment of proteins such as Son of sevenless (SOS) which in turn activate Rat sarcoma virus (RAS).^55^^,^^56^

RAS is able to activate the mitogen-activated protein kinase (MAPK) responsible for the cellular differentiation of osteoclasts and osteoblasts in OP.^55^

Another important molecule is TGFBR2, which codes for transforming growth factor beta (TGFB), a serine/threonine protein kinase. This gene determines the phosphorylation of proteins in the cell nucleus which leads to an increase in the proliferation of osteocytes and osteoblasts.^57^^,^^58^

Insulin-like growth factors (IGF) is a peptide hormone with anabolic properties produced by the liver and by differentiated chondroblasts. IGFs, structurally similar to insulin and responsible for anabolic activities, stimulate the synthesis of aggrecan, type VI and IX collagen and binding proteins for cell proliferation in bone, determining both the quality and the conformation of the bone.^59–62^

## Conclusion

Many pathologies seem multifactorial or simply related to age. I In reality, there are always molecular and cellular imbalances at the basis of these conditions. Unfortunately, management of osteoporosis start too late, only when the pathology is already manifest. Through siRNAs, it is possible to target the molecular bases that lead to OP, to then direct a specific therapy to prevent the actual condition. Various authors have used siRNAs, for example, to identify the target molecules, or as a therapeutic target, or to highlight the efficacy of a given drug. Studies on human cells *in vitro* give us hope for possible future drugs that can combat OP at its origin, without the side effects of current therapies. Appropriate studies are necessary to be able to translate these elegant laboratory studies so that they can be introduced into routine clinical practice.

## Data Availability

The links or identifiers required for the data are present in the manuscript as described.
